# Poxvirus A51R Proteins Negatively Regulate Microtubule-Dependent Transport by Kinesin-1

**DOI:** 10.3390/ijms25147825

**Published:** 2024-07-17

**Authors:** Dahee Seo, Yang Yue, Shin Yamazaki, Kristen J. Verhey, Don B. Gammon

**Affiliations:** 1 Department of Microbiology, University of Texas Southwestern Medical Center, Dallas, TX 75390, USA; 2 Department of Cell and Developmental Biology, University of Michigan Medical School, Ann Arbor, MI 48109, USA; 3 Department of Neuroscience and Peter O’Donnell Jr. Brain Institute, University of Texas Southwestern Medical Center, Dallas, TX 75390, USA

**Keywords:** poxvirus, vaccinia virus, microtubule-associated proteins, microtubule-dependent transport, kinesin-1, virus-host interactions

## Abstract

Microtubule (MT)-dependent transport is a critical means of intracellular movement of cellular cargo by kinesin and dynein motors. MT-dependent transport is tightly regulated by cellular MT-associated proteins (MAPs) that directly bind to MTs and either promote or impede motor protein function. Viruses have been widely shown to usurp MT-dependent transport to facilitate their virion movement to sites of replication and/or for exit from the cell. However, it is unclear if viruses also negatively regulate MT-dependent transport. Using single-molecule motility and cellular transport assays, we show that the vaccinia virus (VV)-encoded MAP, A51R, inhibits kinesin-1-dependent transport along MTs in vitro and in cells. This inhibition is selective as the function of kinesin-3 is largely unaffected by VV A51R. Interestingly, we show that A51R promotes the perinuclear accumulation of cellular cargo transported by kinesin-1 such as lysosomes and mitochondria during infection. Moreover, A51R also regulates the release of specialized VV virions that exit the cell using kinesin-1-dependent movement. Using a fluorescently tagged rigor mutant of kinesin-1, we show that these motors accumulate on A51R-stabilized MTs, suggesting these stabilized MTs may form a “kinesin-1 sink” to regulate MT-dependent transport in the cell. Collectively, our findings uncover a new mechanism by which viruses regulate host cytoskeletal processes.

## 1. Introduction

The microtubule (MT) network functions as an internal “highway” for the intracellular transport of diverse cellular cargo, including vesicles, organelles, and proteins. Motor proteins, such as kinesins and dyneins, are responsible for the intracellular transport of cargo along MT tracks [1]. These motor proteins possess structurally conserved domains called motor domains that bind to MTs and ATP, utilizing energy from ATP hydrolysis to “walk” along MTs [1,2]. While both kinesins and dyneins function as motor proteins, a notable distinction between them lies in the direction of their movement along MTs. Kinesins primarily move cargo toward the plus ends of MTs, typically located near the cell periphery, resulting in anterograde transport [1]. In contrast, dyneins move cargo toward the minus end of MTs, typically found near MT-organizing centers. Therefore, dyneins usually move cargo from the cell periphery to the cell center, also known as retrograde transport [1]. Both motor proteins are composed of multiple subunits, with heavy chain subunits encoding conserved motor domains responsible for establishing physical contact with MTs and powering motor motility, while other subunits govern cargo specificity [3]. While a single dynein motor appears to be responsible for most retrograde transport, with a few exceptional cases, the kinesin protein superfamily consists of approximately 45 members that perform complementary functions [2,4]. These kinesin motors are categorized into ~14 families (kinesin 1–14), with kinesin 1–3 families being the most extensively studied [2,5]. Kinesin-1 is the primary kinesin responsible for most anterograde movement along MTs in eukaryotic cells [6].

Given that MTs are highly dynamic, undergoing phases of polymerization and depolymerization, the formation of stabilized MTs is critical to provide long-lasting MT tracks for cargo transport by motor proteins. Cellular factors such as non-motile MT-associated proteins (MAPs) that directly bind along the MT lattice are critical regulators of MT stability. Moreover, MAPs can have additional effects on MT-dependent transport by either promoting or inhibiting the landing of specific motors onto MTs. For example, human MAP7 recruits kinesin-1 to MTs [7,8,9], while Tau, another human MAP expressed in neurons, inhibits kinesin-1 landing [7,10]. In contrast, both MAP7 and Tau inhibit kinesin-3 landing while having negligible effects on dynein motors [7]. Additionally, MAPs can physically block the movement of motors along MTs. For example, kinesin-1 often detaches from MTs when it runs into Tau on MTs, while dynein motors are only weakly inhibited by Tau [7]. By differentially regulating specific motors, MAPs may control the direction of MT-dependent transport.

Given the critical roles of MTs in facilitating rapid and directional intracellular transport, viruses often usurp the MT network to facilitate their movement to sites of replication and/or to move to the cell periphery for exit [11,12]. Numerous DNA and RNA viruses associate with either dynein and/or kinesin motors to transport their particles around the cell [12]. For example, herpes simplex virus type 1 (HSV-1) and Kaposi’s sarcoma-associated herpesvirus utilize kinesins to transport virions to the cell surface for their release [13,14]. Viruses have also evolved strategies to induce the stabilization of MTs to facilitate their transport. For example, the HSV-1-encoded viral kinase Us3 induces stable MTs near the trans-Golgi network through a mechanism requiring cellular plus-end binding proteins, which bind to the ends of MTs to regulate their stability [15]. This virus-induced MT stabilization enables the efficient anterograde movement of virions and facilitates the spread of infection [15]. Additionally, some viruses, such as the hepatitis B virus, usurp cellular MAPs to form stabilized MTs for their transport [16]. In contrast to the wealth of examples illustrating how viruses promote MT-dependent transport, it is unclear if viruses also encode mechanisms to negatively regulate MT-dependent transport.

Vaccinia virus (VV) is a large DNA virus belonging to the *Poxviridae* family and has been an important model for understanding how viruses manipulate the host cytoskeleton. For example, VV has been shown to hijack kinesin-1 motors to transport a specialized subset of its virions. During the final stages of the poxvirus life cycle, VV virions are released from the host cell to infect neighboring cells. The majority of infectious VV particles (>99%) remain as intracellular mature viruses (IMV), which have a single envelope and can only be released upon cell lysis [17,18]. However, a small fraction of IMVs use kinesin-1 motors to move towards the Golgi where they acquire additional envelopes to form “intracellular enveloped viruses (IEVs)” [18,19]. These IEV particles then use kinesin-1 motors to transit to the cell surface where their outermost envelope fuses with the plasma membrane, releasing them as an extracellular enveloped virus (EEV). The release of EEV particles prior to cell lysis promotes the rapid dissemination of virions to other host cells to initiate new rounds of infection [20,21,22]. However, whether and how VV regulates EEV release remains unclear [23].

Interestingly, VV infection induces the formation of hyperstable subsets of MTs in the cell that are resistant to the MT-depolymerizing drug nocodazole. We previously showed the VV A51R protein to be required for the formation of these stabilized, nocodazole-resistant MTs during infection [24,25]. We demonstrated that VV A51R and other poxvirus-encoded A51R proteins are viral MAPs that directly bind to MTs [24,25]. The binding of VV A51R to MTs promotes their polymerization and inhibits their depolymerization, explaining how these viral factors promote MT stabilization [24].

Given that many cellular MAPs have been shown to regulate MT-dependent transport by kinesins, we asked in the study presented here whether a viral MAP, VV A51R, could also regulate kinesin-mediated transport. Here we show that the binding of kinesin-1 to MTs, the velocity of kinesin-1, and distance traveled along MTs by kinesin-1, are all reduced in the presence of VV A51R. Interestingly, this effect was specific as A51R has minimal effects on kinesin-3 motor function *in vitro*. Importantly, we show that A51R also inhibits kinesin-1- but not kinesin-3-mediated cargo transport in cells. Moreover, the infection of cells with VV strains encoding wild-type (WT) A51R results in the perinuclear accumulation of cellular cargo (lysosomes and mitochondria) that are normally transported to the cell periphery by kinesin-1. In contrast, a VV strain encoding a mutant form of A51R that is unable to bind MTs is unable to cause perinuclear clustering of these organelles. In addition, we show that A51R regulates kinesin-1-dependent IEV transport during infection, leading to a slower release of EEV particles. Using a rigor mutant of kinesin-1 that has reduced MT unbinding rates, we show that these motors can accumulate on a subset of stable MTs bound by A51R, suggesting that A51R-stabilized MTs may create a “kinesin-1 sink”. The selective preference of kinesin-1 for these A51R-stabilized MTs may explain how A51R proteins can have significant effects on MT-dependent transport despite only binding to a subset of MTs. To our knowledge, this establishes VV A51R as the first animal virus-encoded protein shown to negatively regulate the kinesin-dependent transport of cellular and viral cargo and reveals a new mechanism by which viruses manipulate the host cytoskeleton.

## 2. Results

### 2.1. A51R Preferentially Inhibits Kinesin-1 Function In Vitro

VV A51R directly binds to, stabilizes, and bundles MTs in cells, exhibiting classic properties of a MAP [24]. The MT bundling by A51R likely results from its ability to self-interact, wherein the C-terminal MT-binding domain of each A51R monomer can engage with separate MTs and thus bridge these individual MTs [24]. While we previously found purified A51R proteins to primarily form dimers in solution [24], in this study, we used super-resolution microscopy to investigate if A51R formed filaments of uniform or various lengths along MT tracks in cells. Interestingly, we found A51R to form long filamentous structures along MT tracks that ranged from 0.2–4 μm in length during infection, suggesting that A51R oligomerization in cells may result in filaments that coat MT tracks to varying degrees (Figure S1).

**Figure 1:**
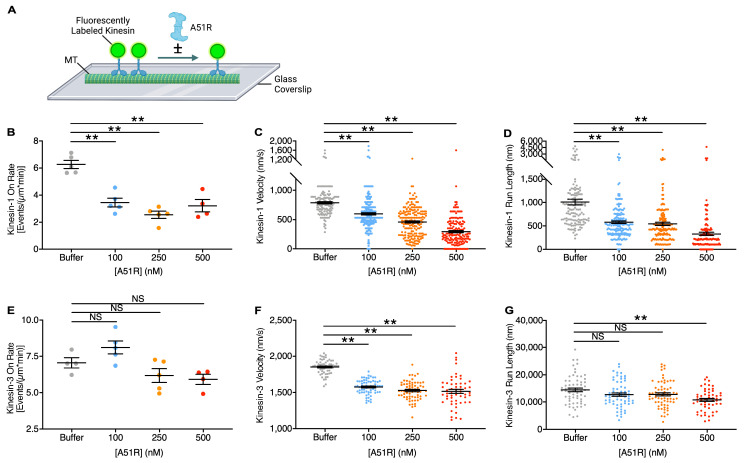
A51R preferentially inhibits kinesin-1 function *in vitro*. (**A**) Schematic overview of total internal reflection fluorescence (TIRF) microscopy-based in vitro single-molecule motility assays with fluorescent kinesins. The image was created with Biorender.com. (**B**–**D**) On-rate (**B**), velocity (**C**), and run length (**D**) for kinesin-1 [KIF5C(1-560)-3xmCit] with in vitro single-molecule assays in the presence or absence (buffer control) of A51R. Horizontal lines in dot-plots are means (±SE). One-way ANOVA followed by Dunnett’s post-test. ** = *p* < 0.01, NS = not significant. A total of at least 4, 136, and 136 individual measurements were made for each treatment group to calculate on-rate, velocity, and run length, respectively. (**E**–**G**) On-rate (**E**), velocity (**F**), and run length (**G**) for kinesin-3 [KIF1A(1-393)-3xmCit] with in vitro single-molecule assays in the presence or absence (buffer control) of A51R. Horizontal lines in dot-plots are means (±SE). One-way ANOVA followed by Dunnett’s post-test. ** = *p* < 0.01, NS = not significant. A total of at least 4, 55, and 55 individual measurements were made for each treatment group to calculate on-rate, velocity, and run length, respectively.

Given that MT-bound cellular MAPs can either promote or inhibit kinesin binding to MTs, we examined if A51R binding could alter kinesin binding to, and/or movement along, MTs. We were particularly interested in testing if A51R affects kinesin-1 motors because IEV particles have been shown to use these motors to move along MTs during VV infection [22,23]. Moreover, kinesin-1 is the primary kinesin responsible for most anterograde movement along MTs in eukaryotic cells [6]. Therefore, we tested the effect of the recombinant, purified His-tagged VV A51R (His-A51R) protein [24] on kinesin-1 function *in vitro*, using total internal reflection fluorescence (TIRF) microscopy-based single-molecule motility assays [26,27,28]. We used a constitutively active form of the kinesin-1 heavy chain KIF5C fused to three tandem mCitrine tags [KIF5C(1-560)-3xmCit] and monitored its movement along *in vitro*-polymerized taxol-stabilized MTs labeled with HiLyte Fluor-647 and bound to a glass coverslip (Figure 1A) [29]. To determine if any effects of A51R on kinesin-1 motors were specific, we also determined A51R effects on a constitutively active form of the kinesin-3 KIF1A tagged with 3xmCit [KIF1A(1-393)-3xmCit]. We then analyzed kinesin landing (on-rate), rate of movement (velocity), and distance traveled (run length) on MTs in the absence or presence of varying concentrations of His-A51R.

We found the on-rate, velocity, and run length of kinesin-1 to all significantly decrease with His-A51R concentrations of 100–500 nM (Figure 1B–D and Figure S2). For example, at the highest concentration of His-A51R, the mean kinesin-1 on-rate, velocity, and run length decreased to only ~50%, 38%, and 33%, respectively, of the control (buffer) group. Importantly, prior single-molecule motility assays estimated kinesin-1 velocity and run lengths to be ~700–800 nm/s and ~830 nm, respectively [30,31]. These results are remarkably similar to our mean kinesin-1 velocity (~750 nm/s) and run lengths (~1000 nm) under our control (buffer) conditions, suggesting our motors were fully active.

In contrast to our kinesin-1 results, the on -rate of kinesin-3 was not significantly affected at any concentration of His-A51R (Figure 1E). Although all concentrations of His-A51R decreased kinesin-3 velocity along MTs, these effects were relatively modest. For example, at the highest concentration of His-A51R, kinesin-3 velocity was still ~82% of the velocity observed in control conditions where His-A51R was absent (Figure 1F). There were also marginal effects on kinesin-3 run length, with only the highest concentration of His-A51R significantly reducing the run length to ~75% of the distance traveled in the absence of His-A51R (Figure 1G). It is important to note that the overall higher velocities and run lengths of kinesin-3 versus kinesin-1 in control (buffer) conditions are consistent with prior comparisons of these motors [30,32]. Collectively, these data suggest that A51R preferentially inhibits kinesin-1 binding and movement along MTs.

### 2.2. A51R Specifically Inhibits Kinesin-1-Dependent Cargo Transport in Cells

**Figure 2:**
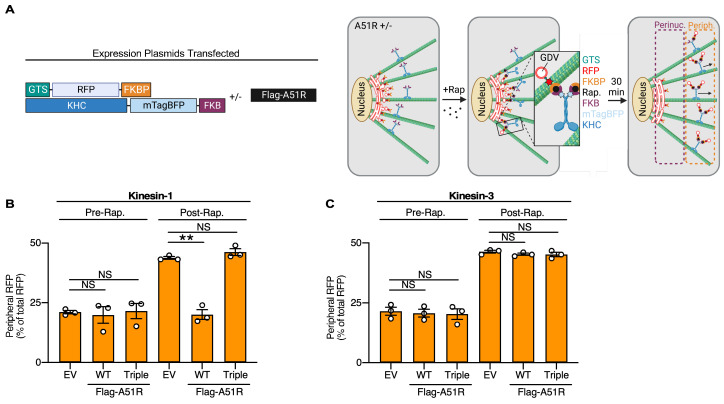
A51R specifically inhibits kinesin-1-dependent cargo transport in cells. (**A**) Schematic overview of Golgi dispersion assay in U2OS cells showing plasmid constructs (left) and rapamycin-induced dispersion of Golgi-derived vesicles by transfected kinesin (right). Rap., Rapamycin. The image was created with Biorender.com. (**B**) Kinesin-1 (KIF5C) Golgi dispersion assay in U2OS cells co-transfected with empty vector (EV), FA51R wild-type (WT), or FA51R^Triple^ mutant (Triple) expression plasmids. Data are means (±SE). Unpaired two-tailed Student’s *t*-test. ** = *p* < 0.01, NS = not significant. (**C**) Kinesin-3 (KIF1A) Golgi dispersion assay in U2OS cells co-transfected with empty vector (EV), FA51R wild-type (WT), or FA51R^Triple^ mutant (Triple) expression plasmids. Data are means (±SE). Unpaired two-tailed Student’s *t*-test. NS = not significant.

To determine if A51R also inhibits kinesin-1 function in cells, we used a Golgi dispersion assay, which allows one to track the anterograde movement of Golgi-derived vesicles by specific kinesin motors (Figure 2A) [33]. In this assay, cells are co-transfected with plasmids expressing a constitutively active, fluorescently tagged KHC fused with an FKBP-rapamycin-binding (FRB) domain, and a red fluorescent protein (RFP) fused to a Golgi-targeting sequence (GTS) and a FKBP domain (Figure 2A). The FRB and FKBP domains derive from the FKBP-rapamycin-FRB heterodimerization system, in which FKBP and FRB domains heterodimerize in the presence of the drug rapamycin [33,34]. Thus, once rapamycin is added to cells, FRB-tagged kinesins heterodimerize with FKBP-RFP-GTS (residing in Golgi membranes) and transport Golgi-derived vesicles along MTs toward the plasma membrane. To assess the movement of kinesins in cells, we fixed co-transfected cells after rapamycin treatment and analyzed the ratio of RFP signal in perinuclear areas (e.g., Golgi) to that in peripheral regions (e.g., plasma membrane-adjacent). Only cells with nuclei predominantly on one side of the cell were analyzed so that the major cytoplasmic space between the nuclear membrane and the plasma membrane could be equally divided into “perinuclear” and “peripheral” regions (Figure 2A). To determine if VV A51R–MT interactions affected kinesin-dependent transport to the cell periphery, we also co-transfected cells with plasmids expressing Flag-tagged A51R (FA51R), “triple” mutant FA51R (FA51R^Triple^), which encodes three amino acid substitutions (R275A/K295A/K302A) that destroy A51R–MT interactions [24], or an empty vector (EV) as a control (Figure 2A).

As expected, RFP signals remained mostly in perinuclear regions before rapamycin treatment, reflecting the GTS-mediated association of RFP with the Golgi apparatus (Figure 2B and Figure S3). However, after rapamycin treatment, significantly more RFP signal was observed in the periphery in EV-transfected cells as RFP-labeled, Golgi-derived vesicles move by kinesin-1 to the cell periphery (Figure 2B and Figure S3). Consistent with our *in vitro* studies, FA51R expression from plasmids significantly inhibited the kinesin-1-dependent transport of vesicles, indicated by lower RFP signals in the periphery of FA51R-transfected cells (Figure 2B and Figure S3). However, the FA51R^Triple^ expression vector did not inhibit kinesin-1-dependent movement of vesicles to the cell periphery (Figure 2B and Figure S3), suggesting that A51R–MT interactions are required to block kinesin-1-dependent transport in cells.

When the same assay was performed with kinesin-3 instead of kinesin-1, we did not observe a significant effect of FA51R or FA51R^Triple^ expression on anterograde movement of RFP-labeled vesicles after rapamycin treatment (Figure 2C and Figure S3), consistent with our *in vitro* studies that suggested A51R only weakly inhibits kinesin-3 function. Collectively, these data suggest that A51R specifically inhibits kinesin-1-dependent transport in cells in a manner that requires an A51R–MT interaction.

### 2.3. A51R–MT Interactions Alter the Localization of Endogenous Cellular Cargo during Infection

A previous report showed that VV infection induces the clustering of lysosomes and mitochondria in perinuclear areas during infection of HeLa cells [35]. It was suggested that this phenotype may arise because VV may somehow impede the anterograde transport of these organelles to the cell periphery [35]. Given that kinesin-1 is the major motor driving anterograde transport of mitochondria and lysosomes [36,37,38], we wondered whether A51R may be responsible for the perinuclear accumulation of these organelles. Therefore, we monitored the localization of these cellular organelles over time during infection using VV strains: ΔA51R^FA51R^ and ΔA51R^FA51RTriple^. ΔA51R^FA51R^ is a recombinant VV strain expressing WT FA51R under the *A51R* gene promoter, whereas the ΔA51R^FA51RTriple^ strain expresses the MT-interaction-deficient FA51R^Triple^ mutant protein [24].

**Figure 3:**
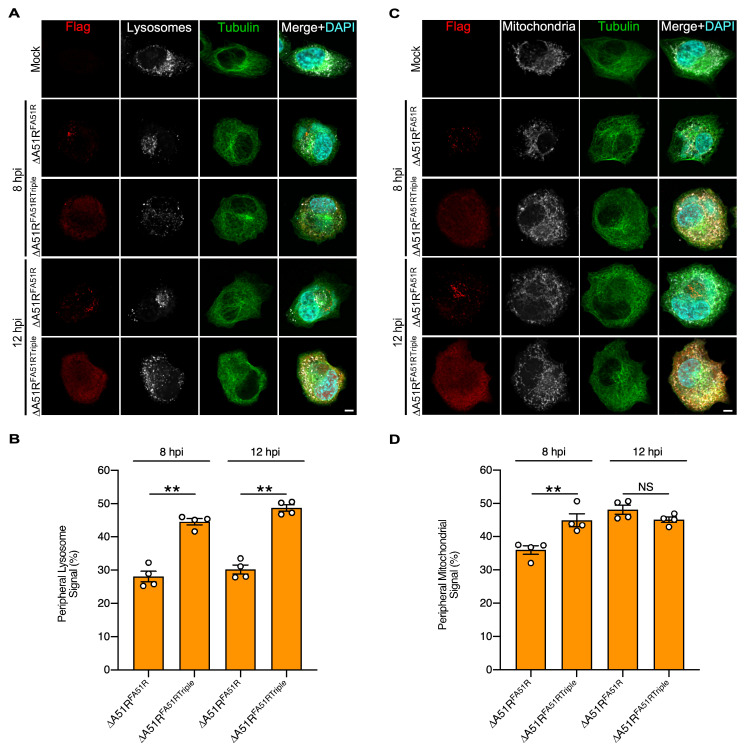
A51R–MT interactions alter the localization of endogenous cellular cargo during infection. (**A**) Representative lysotracker images in mock- ∆A51R^FA51R^- and ∆A51R^FA51RTriple^-infected HeLa cells at 8 and 12 hpi. Scale bar, 5 μm. (**B**) Distribution of lysotracker signal from (**A**) comparing ∆A51R^FA51R^- and ∆A51R^FA51RTriple^-infected cells. (**C**) Representative mitotracker images in mock- ∆A51R^FA51R^- and ∆A51R^FA51RTriple^-infected HeLa cells at 8 and 12 hpi. Scale bar, 5 μm. (**D**) Distribution of mitotracker signal in cells from **C**. Data are means (±SE). Unpaired two-tailed Student’s *t*-test. ** = *p* < 0.01, NS = not significant.

HeLa cells were mock-infected or infected with ΔA51R^FA51R^ or ΔA51R^FA51RTriple^, then stained with a lysotracker to visualize lysosome localization using confocal microscopy. We then quantified the percentage of lysosomes in perinuclear and peripheral regions of a cell, using a similar strategy as described for the Golgi dispersion assay. In ΔA51R^FA51RTriple^-infected cells, lysosomes typically displayed a “dispersed” pattern, with roughly equal staining found in both peripheral and perinuclear areas of the cytoplasm at 8 or 12 h-post infection (hpi). However, when cells were infected with ΔA51R^FA51R^, there was significantly more lysosome accumulation near the perinuclear region of infected cells (Figure 3A,B). We also used mitotracker to observe mitochondria localization during infection. At 8 hpi, there were significantly higher levels of mitochondria in the perinuclear region of ΔA51R^FA51R^- versus ΔA51R^FA51RTriple^-infected cells. However, at 12 hpi, cells infected either with A51R^FA51R^ or ΔA51R^FA51RTriple^ displayed more dispersed mitochondria staining patterns, consistent with previous observations by Schepis et al. [35], where they observed only transient re-localization of mitochondria to perinuclear regions during the early stages of infection (Figure 3C,D). These data suggest that A51R–MT interactions play a role in the perinuclear clustering of organelles previously observed during VV infection.

### 2.4. A51R Inhibits EEV Release

**Figure 4:**
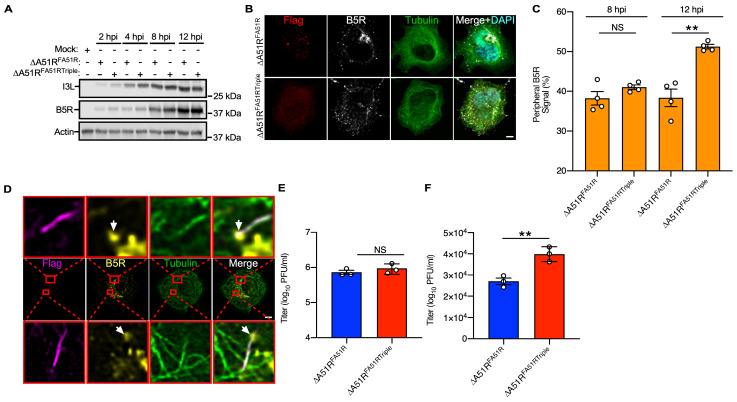
A51R inhibits IEV movement to the periphery and EEV release. (**A**) Immunoblot of U2OS whole-cell extract (WCE) infected with the indicated VV strains (MOI = 3) for indicated times. I3L and B5R mark early and late VV proteins, respectively. (**B**) Representative confocal microscopy images of VV-infected U2OS cells 12 hpi. Scale bar, 5 μm. (**C**) Distribution of B5R signal in infected U2OS cells from (**B**). Data are means (± SE). Unpaired two-tailed Student’s *t*-test. ** = *p* < 0.01, NS = not significant. (**D**) Representative super-resolution microscopy image of U2OS cell infected with ∆A51R^FA51R^ for 18 h and showing FA51R (Flag), tubulin, and B5R (marker for IEV) staining. Scale bar, 5 μm. A zoomed-in image of the area indicated with the red box is shown to the right. White arrows indicate IEV particles on A51R-bound MTs. (**E**,**F**) Total (cells + supernatant) titer **(E**) and EEV titer in the supernatant (**F**) of U2OS cell cultures infected (MOI = 3) with the indicated strains for 24 h. EEV titer was determined after MV neutralization. Data are means (±SE). Unpaired two-tailed Student’s *t*-test. ** = *p* < 0.01, NS = not significant.

Next, we wanted to test if A51R–MT interactions affect IEV transport and subsequent EEV release during infection because IEV transport along MTs using kinesin-1 to the cell surface and then are released as EEV [22,39]. First, we wanted to use immunofluorescence (IF) staining of VV B5R, which is specifically incorporated into IEV particles [40], to analyze the distribution of IEV in infected U2OS cells. Virion formation of VV typically initiates ~8 hpi [35], and consistent with this, we observed an increase in B5R expression by 8 hpi (Figure 4A). To determine if A51R–MT interactions affected IEV distribution within infected cells, we used a similar strategy as in Figure 2 to determine the proportion of B5R staining that is perinuclear or peripheral at 8 hpi and 12 hpi with either ΔA51R^FA51R^ or ΔA51R^FA51RTriple^ VV strains. Importantly, B5R levels were similar during ΔA51R^FA51R^ and ΔA51R^FA51RTriple^ infections (Figure 4A), ensuring that differences in B5R staining distribution cannot be due to expression levels.

Our data suggest that B5R in both ΔA51R^FA51R^ or ΔA51R^FA51RTriple^ infections is virtually all perinuclear at 8 hpi as virion formation initiates. By 12 hpi, B5R staining begins to shift into peripheral regions as IEV moves away from perinuclear assembly sites (Figure 4B,C). At this later time point, peripheral B5R signals were significantly higher in ΔA51R^FA51RTriple^-infected cells than in ΔA51R^FA51R^-infected cells (Figure 4C), suggesting that A51R–MT interactions are required to impede the kinesin-1-dependent transport of IEVs to the periphery. To further confirm if this differential localization of IEV particles is due to the lack of A51R–MT interactions, we conducted a separate set of experiments using ΔA51R, a VV strain with the entire *A51R* gene deleted (Figure S4) [25]. We observed a similar difference in B5R signal localization between ΔA51R-infected cells and ΔA51R^FA51R^-infected cells (Figure S4), suggesting that A51R blocks kinesin-1-dependent IEV transport towards the cell surface. Additional support for this possibility came from super-resolution imaging of ΔA51R^FA51R^-infected cells where IEV particles (marked by B5R staining) could often be observed on MTs that were also coated with FA51R, suggesting IEV particles move along MT tracks that are bound by A51R (Figure 4D).

It is important to note that although B5R staining may often represent IEV particles, some B5R staining, particularly in perinuclear areas, may reflect B5R protein not yet incorporated into fully infectious IEV particles. Therefore, in order to corroborate our B5R localization studies, we asked if A51R–MT interactions also influence EEV release from infected cells. We previously reported no difference in the replication of ΔA51R^FA51R^ and ΔA51R^FA51RTriple^ strains in U2OS cells [24]. Given that these prior experiments measured total VV titers from both cells and supernatants (and thus were a mixture of both IMV and EEV particles), here we wanted to also specifically measure EEV titers to determine if A51R–MT interactions influence the release of EEV. It is important to note that EEV can be specifically titered by treating collected supernatants from infected cultures with IMV-neutralizing antibodies [25]. Thus, U2OS cells were infected cells under a high multiplicity of infection (MOI) conditions with ΔA51R^FA51R^ or ΔA51R^FA51RTriple^ strains, and then both total VV total (IMV + EEV) titers and EEV-specific titers were measured. While total titers were similar between ΔA51R^FA51R^- and ΔA51R^FA51RTriple^-infected cultures, as we previously observed [24] (Figure 4G), EEV titers were significantly higher during ΔA51R^FA51RTriple^ infection (Figure 4H), suggesting that A51R–MT interactions hinder EEV release.

### 2.5. Kinesin-1 Is Preferentially Recruited to A51R-Bound MTs

Tubulin post-translational modifications (PTMs), such as acetylation and detyrosination, are often found on stable populations of MTs in the cell. Prior work has suggested that kinesin-1 preferentially binds to these stabilized MT populations because (1) kinesin-1 may recognize these PTMs; (2) these PTMs alter MAP–MT interactions, which then influence kinesin-1 binding; or (3) these modifications simply accumulate on long-lived, stable MT tracks on which kinesin-1 is more likely to bind [30,41,42]. However, recent studies suggest that kinesin-1 binding to MTs is likely more dependent upon MT stability than the presence of these PTMs [43]. Thus, we were interested in determining if A51R-bound MTs are typically modified by tubulin PTMs because A51R-bound MTs are hyperstable and resistant to depolymerization with nocodazole [24]. Therefore, we investigated whether A51R exhibits preferential binding to MT filaments with tubulin PTMs associated with MT stabilization. After infecting cells with ΔA51R^FA51R^, we stained them for post-translationally modified tubulin (either acetylation or detyrosination) and examined whether A51R specifically co-localizes with MTs containing these PTMs using super-resolution and stimulated emission depletion (STED) microscopy, which allowed us to visualize individual MT filaments at a high resolution. Using both microscopy methods, we found that A51R preferentially binds to MTs that are not acetylated during infection (Figure S5A,B). However, A51R was typically associated with MT filaments and bundles displaying detyrosination signal (Figure S5C,D), suggesting that A51R either preferentially binds detyrosinated MTs or that A51R-stabilized MTs are more likely to become detyrosinated.

**Figure 5:**
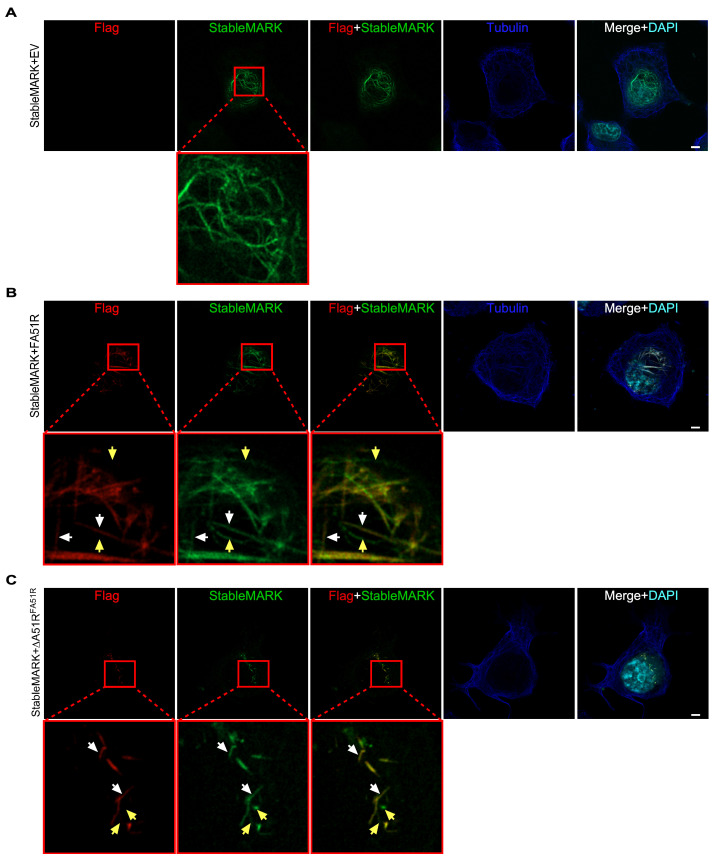
Kinesin-1 is preferentially recruited to A51R-bound MTs. (**A**,**B**) Super-resolution microscopy images of U2OS cells co-transfected with StableMARK and empty pCDNA3 (EV) expression plasmids (**A**) or with StableMARK and WT FA51R pCDNA3 expression plasmids (**B**). Images taken 24 h post-transfection. (**C**) Super-resolution microscopy image of ∆A51R^FA51R^-infected U2OS cells transfected with a construct expressing StableMARK 24 hpi. Zoomed-in images of the areas indicated are shown with red boxes. White arrows indicate examples of overlap between Flag (FA51R) and StableMARK signal while yellow arrows indicate StableMARK signal lacking clear overlap with FA51R. Scale bar = 5 μm.

To directly determine if kinesin-1 motors are recruited to the stabilized MTs bound by A51R, we transfected cells with a plasmid expressing mNeonGreen-tagged kinesin-1 heavy chain (a.a. 1-560) encoding a G234A substitution. This construct is also known as “StableMARK” [43]. The G234A substitution perturbs kinesin-1 ATPase activity and results in a kinesin-1 rigor mutant that has a very low rate of MT unbinding [44], allowing easier visualization of MTs that kinesin-1 may preferentially bind to since, unlike WT kinesin-1, which is more prone to release from MTs after hitting “road blocks” such as MAPs, this rigor mutant remains tightly bound to the MT lattice [43]. When StableMARK was expressed with an empty vector (control conditions), we found it to co-localize with a subset of perinuclear MT tracks, as previously described (Figure 5A) [43]. However, when we co-transfected StableMARK and A51R expression plasmids together, we found StableMARK to accumulate on A51R-decorated MTs within the cells (Figure 5B). This recruitment of StableMARK to A51R-bound MTs was also observed during ΔA51R^FA51R^ infection (Figure 5C). Collectively, these data suggest that kinesin-1 rigor mutants may preferentially bind to the MT tracks bound by A51R, possibly because these MTs are likely to be long-lived and stable [30,41].

## 3. Discussion

Cellular MAPs are thought to act as “traffic cops” that can either promote or inhibit motor movement along MTs, thereby controlling overall intracellular transport directionality by specific motors. For example, Tau inhibits kinesin-1 movement along MTs while MAP7 promotes kinesin-1-dependent transport [7,8,9]. Interestingly, dysregulation of MT-dependent transport is a hallmark of many neurodegenerative disorders characterized by abnormal Tau function (termed “tauopathies”) [45,46,47,48], illustrating the need for proper intracellular transport regulation in the cell. Prior studies have shown that several viruses promote MT stabilization to usurp MT-dependent transport to facilitate their movement to sites of replication upon entry or for their escape from the cell after replication has occurred [12]. However, our study now provides evidence that an animal virus-encoded MAP can negatively regulate MT-dependent transport, illustrating that the regulation of intracellular transport machinery by viruses is more complex than originally thought.

**Figure 6:**
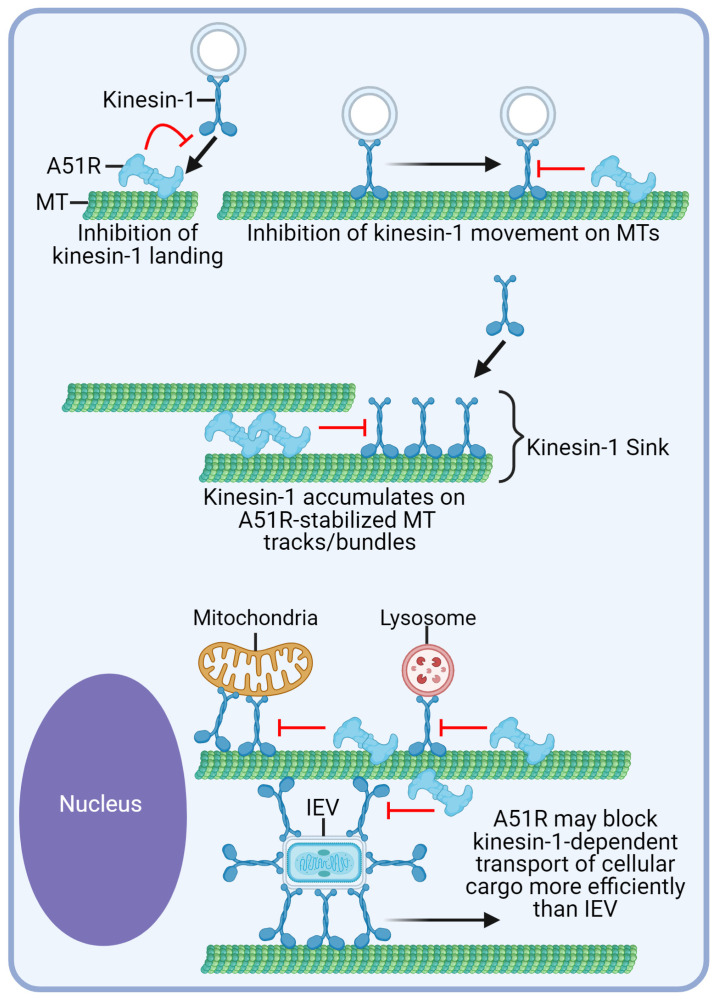
Model of A51R-mediated negative regulation of kinesin-1-dependent transport. A51R self-interaction [24] allows the formation of A51R filaments that may impede the landing of kinesin-1. However, kinesin-1 motors that land on areas of the MT lattice not coated with A51R can still have their movement inhibited by A51R “road blocks”. A51R-mediated MT stabilization and bundling [24] creates long-lived MTs to which kinesin-1 is attracted, forming a “kinesin-1 sink”. Movement of IEV particles may be less hindered than cellular cargo due to the denser and broader distribution of kinesin-1 motors on IEV particles [19], allowing IEVs to switch to MT tracks after encountering A51R molecules. Figure was created with Biorender.com.

We found A51R to preferentially inhibit kinesin-1 landing and movement along MTs *in vitro* and to block kinesin-1-dependent trafficking of cargo in cells. This effect was relatively specific to kinesin-1, given that A51R had no effect on the landing rate of kinesin-3 and minimal effects on kinesin-3 movement along MTs *in vitro*. We also observed no detectable effect on kinesin-3-dependent transport in cells. Interestingly, cellular MAPs have also been shown to have specificity in the kinesins they regulate, with some inhibiting kinesin-1, but not kinesin-3 [49]. Why A51R was unable to inhibit kinesin-3 landing and had minimal effects on kinesin-3 movement along MTs remains unclear. However, prior studies have shown that kinesin-3 motors encode a large, positively charged “K loop” that is unique to this subfamily of motors, leading to an increased affinity of kinesin-3 motors for MTs compared to kinesin-1 [28,32]. Furthermore, kinesin-3 motors have been termed “superprocessive” because they exhibit greater run lengths before dissociating from MTs than kinesin-1 motors [28,32]. Thus, A51R may be unable to outcompete kinesin-3 motors for binding to MTs, and once bound, kinesin-3 motors may be less likely to dissociate when encountering MT-bound A51R proteins. In cells, kinesin-1 motors preferentially bind stable, long-lived MT tracks, whereas kinesin-3 motors are typically associated with more dynamic MTs; thus, in cells, kinesin-3 may avoid A51R-bound MTs [30,41]. We hypothesize that A51R-stabilized MTs may, in effect, create a “kinesin-1 sink” by creating long-lived MT tracks that kinesin-1 can land on. While, at first, this model may seem incongruent with our in vitro studies showing A51R can inhibit kinesin-1 landing, it is important to note that the concentration of A51R used in these in vitro experiments may lead to more complete decoration of MTs (providing fewer kinesin-1 binding sites) than in cells. Indeed, our immunofluorescence experiments show that A51R only binds to a subset of MTs in cells and we often see A51R only coat a fraction of MT tracks it binds to (e.g., Figure 4D). Thus A51R-stabilized MTs in cells are more likely to have sites available for kinesin-1 binding. Thus, we believe that the main mode of A51R-mediated regulation of kinesin-1 transport is to act as a roadblock to motor motility. Indeed, our observation of IEV particles on A51R-bound MTs (Figure 4D) and the accumulation of StableMARK kinesin-1 rigor motors on A51R-bound MTs (Figure 5B,C) is consistent with this. If kinesin-1 in fact has a preference for the stabilized MTs tracks created by A51R, this may explain how this viral MAP, which only localizes to a subset of MTs, can still significantly affect kinesin-1-dependent transport in cells (Figure 6).

The specificity of A51R for kinesin-1 was surprising since this motor protein transports IEVs, the key type of VV virion, to the surface of infected cells to be released as EEVs [22,23]. Although we observed ΔA51R^FA51RTriple^-infected cells to release significantly more EEV than ΔA51R^FA51R^-infected cells, the ~1.5-fold increase in EEV release in mutant virus infections did not result in overall differences in VV spread and replication in these cells, as evidenced by the indistinguishable replication kinetics of these two viruses in U2OS cells [24]. In animals, EEV particles are important for long-range dissemination of VV to distal sites [21]. However, EEV particles can also trigger host immune responses in vivo [21]. Thus, the proper timing (and level) of EEV release may be critical for VV immune evasion, and thus the relatively minor increase in EEV release by ΔA51R^FA51RTriple^-infected cells may have more dire consequences *in vivo*. Consistent with this, we found ΔA51R^FA51RTriple^ strains to be highly attenuated in their virulence in mice compared to the ΔA51R^FA51R^ strain [24].

It is possible that A51R may serve as a greater impediment to the kinesin-1-dependent transport of cellular cargo than IEV particles. Indeed, a recent study demonstrated that IEVs recruit and distribute an average of ~320 kinesin-1 molecules across their surface, as opposed to cellular cargo that typically exhibits fewer kinesin motors concentrated at specific locations [19]. Therefore, the broad distribution of kinesin-1 motors across the surface of IEVs may enhance their ability to navigate through the MT network by allowing IEV particles to switch MT tracks or bypass potential obstacles, such as A51R, during their movement on MTs (Figure 6) [19]. We speculate that the difference in the number of kinesin-1 molecules may also explain why lysosome transport is significantly more inhibited over a prolonged period during infection compared to mitochondria. Mitochondria, being larger organelles with more surface area to bind motor proteins, are more likely to bypass A51R-bound MTs. Why A51R-mediated inhibition of organelle movement may be beneficial to VV is unclear, but one possibility is that by blocking kinesin-1 function early in infection, the VV may keep mitochondria in close proximity to its perinuclear viral factories in order to ensure an ample supply of ATP for its replication-related processes.

In conclusion, we demonstrate that VV A51R hinders kinesin-1 function both in vitro and in cells, similar to the activities exhibited by cellular MAPs such as Tau and MAP4 [50]. Our study unveils the first instance of a viral MAP that can negatively regulate kinesin-1-dependent transport along MTs, uncovering a new function for viral MAPs.

### Limitations of the Study

A51R antibodies are currently unavailable, necessitating the use of His- or Flag-tags for A51R protein purification and/or detection. We demonstrated that His-tagged mCherry or His-tagged A51R Triple mutant proteins cannot bind MTs in vitro, suggesting His tags do not mediate A51R-MT interactions in our in vitro experiments [24]. However, the possibility that His tags have minor effects on A51R structure/function cannot be ruled out. A similar point must be stressed for our *in cellulo* experiments using Flag-tagged A51R constructs. We have shown that the replication defect of the ∆A51R strain can be rescued by insertion of the Flag-tagged A51R open reading frame back into the ∆A51R strain (creating ∆A51R^FA51R^) and that the ∆A51R^FA51R^ replicates similar kinetics and yields as wild-type VV (WR strain) [25] and retains virulence in mice [24]. Thus, while these data indicate that Flag-A51R reconstitutes phenotypes observed with VV encoding non-tagged A51R, epitope tag effects on A51R structure/function cannot be ruled out. Finally, due to a lack of live-cell imaging tools needed for time course assays, we were unable to determine if A51R preferentially binds detyrosinated MTs or whether A51R-stabilized MTs become preferentially detyrosinated over time.

## 4. Materials and Methods

### 4.1. Cell and Virus Culture

U2OS and HeLa cells were maintained in DMEM supplemented with 10% FB Essence (FBE) and BSC-40 cells were cultured in MEM supplemented with 5% FBE. All of the above cell media was additionally supplemented with 1% Antibiotic/Antimycotic, 1% L-Glutamine, and 1% non-essential amino acids. COS-7 were cultured in DMEM with 10% Fetal Clone III and 1% GlutaMAX. All mammalian cells were maintained at 37 °C in a 5% CO_2_ atmosphere.

VV stocks were amplified and titrated on BSC-40 cells as described [25]. EEV titers were determined after neutralization with L1R antibodies as described [25]. Virus infections were carried out for 2 h in serum-free DMEM at 37 °C. After 2 h, innocula were aspirated and normal growth medium was added to each well. All virus strains used for this study are listed in Table S1.

### 4.2. Plasmid Construction

The VV FA51R and FA51R^Triple^ pcDNA3 plasmids have been previously described in detail elsewhere [25]. The VV His-A51R expression construct has been described [24]. Briefly, the A51R ORF was PCR-amplified from FA51R pCDNA3 vectors using primers encoding an N-terminal His-tag and NcoI/NotI cut sites and the resulting products were ligated into a modified pET22b vector in which the pelB sequence was removed.

For single-molecule motility assays, plasmids encoding constitutively active versions of the kinesin-1 motor KIF5C (aa 1-560) and the kinesin-3 motor KIF1A (aa 1-393 followed by the leucine zipper of GCN4) were used and have been described [30,32]. Motors were tagged with three tandem mCitrine (mCit) fluorescent proteins for single-molecule imaging.

Plasmids used in the Golgi dispersion assay have been previously described [51]. Briefly, truncated, constitutively active domains of kinesin heavy chains were cloned to generate the tagged kinesin motor constructs. For kinesin-1 (KIF5C), rat KIF5C a.a. 1-560 was cloned, and for kinesin-3 (KIF1A), rat KIF1A a.a 1-393 fused to a leucine zipper domain (for dimeric motor formation) was cloned and inserted into the expression vector in-frame with C-terminal fluorescent protein tags (mTagBFP) and FRP domains. The Golgi targeting GMAP-mRFP-FKBP construct has been previously described [27,33]. The expression and activity of these constructs have been previously shown [27,33,51].

### 4.3. Expression and Purification of His-A51R

Recombinant His-tagged A51R was expressed and purified as previously described [24]. Briefly, His-A51R pET22b vectors were transformed into BL21(DE3) *E. coli* cells and then grown in Luria broth at 37 °C, induced at mid-log phase with IPTG (0.5 mM), and then harvested by centrifugation (5000 rpm, 20 min, 4 °C) after 4 h. Pellet were then lysed by sonication in Buffer A (50 mM Tris-HCl pH 7.4, 500 mM NaCl, 10% glycerol, 50 mM imidazole, 4 °C). Soluble fractions were obtained by centrifugation, and supernatants were loaded onto a HisTrap HP 1ml column, washed, and eluted with Buffer B (50 mM Tris-HCl pH 7.4, 500 mM NaCl, 500 mM imidazole, 10% glycerol). Pooled His-A51R factions were run over a Superdex 200 Increase 10/300 size-exclusion chromatography (SEC) column (GE Healthcare), exchanged into Storage Buffer (50 mM Tris-HCl pH 7.4, 500 mM NaCl, 10% glycerol), concentrated, and snap frozen prior to storage at −80 °C.

### 4.4. Immunoblotting

For immunoblots, the cells were transfected or infected as described and harvested in Reporter Lysis Buffer (Promega, Madison, WI, USA). Cells were lysed by freeze–thaw three times, and the protein extracts were boiled for 10 min prior to SDS-PAGE electrophoresis at 50 V for approximately 4 h. Separated proteins were transferred in Towbin Buffer (BioRad, Hercules, CA, USA) onto nitrocellulose membranes at 150 mA at 4 °C for 90 min and blocked with Odyssey Blocking Buffer (LI-COR, Lincoln, NE, USA) for 1 h at RT. Membranes were blotted with primary antibodies overnight at 4 °C, with actin serving as a loading control. After 3 × 5 min PBS-T (PBS, 0.1% Tween) washes, membranes were incubated in secondary antibodies conjugated to an IRDye (LI-COR) for 1 h, washed 3 × 5 min in PBS-T, and then underwent a final 5 min PBS wash. Membranes were developed with a LI-COR Odyssey Fc Imager.

### 4.5. Single-Molecule Motility Assays

Single-molecule kinesin motility assays have been previously described [26,27,28]. Briefly, COS-7 cells were transfected with Trans-IT LT1 (Mirus) according to the manufacturer’s instructions to express 3xmCit-tagged kinesin motors. COS-7 cells were harvested 24 h after transfection by low-speed centrifugation at 4 °C. The pellet was rinsed once in PBS and resuspended in ice-cold lysis buffer (25 mM HEPES/KOH, 115 mM potassium acetate, 5 mM sodium acetate, 5 mM MgCl_2_, 0.5 mM EGTA, and 1% Triton X-100, pH 7.4) freshly supplemented with 1 mM ATP, 1 mM PMSF, and 1% (vol/vol) protease inhibitors. After the lysate was clarified by centrifugation at full speed at 4 °C, aliquots of the supernatant were snap-frozen in liquid nitrogen and stored at −80 °C until further use.

HiLyte647-labeled MTs were polymerized from purified tubulin including 10% Hilyte647-labeled tubulin (Cytoskeleton) in BRB80 buffer (80 mM Pipes/KOH pH 6.8, 1 mM MgCl_2_, and 1 mM EGTA) supplemented with 1 mM GTP and 2.5 mM MgCl_2_ at 37 °C for 30 min. Then, 20 μM of taxol in prewarmed BRB80 buffer was added and incubated at 37 °C for an additional 30 min to stabilize MTs. These MTs were stored in the dark at room temperature for further use. A flow cell (~10 μL volume) was assembled by attaching a clean #1.5 coverslip to a glass slide with two strips of double-sided tape. Polymerized MTs were diluted in BRB80 buffer supplemented with 10 μM taxol and then were infused into flow cells and incubated for 5 min at RT for adsorption onto coverslips. Subsequently, blocking buffer (15 mg/mL BSA and 10 μM taxol in P12 buffer) was infused and incubated for 5 min. Finally, 0.5 μL cell lysate expressing 3xmCi-tagged kinesin motor and indicated concentrations of unlabeled His-A51R protein in motility mixture [2 mM ATP, 0.4 mg/mL casein, 6 mg/mL BSA, 10 μM taxol, and oxygen scavenging (1 mM DTT, 1 mM MgCl_2_, 10 mM glucose, 0.2 mg/mL glucose oxidase, and 0.08 mg/mL catalase) in P12 buffer] were added and the flow cell was sealed with molten paraffin wax.

Images were acquired by TIRF microscopy using an inverted microscope Ti-E/B (Nikon, Mellville, NY, USA) equipped with a perfect focus system (Nikon), a 100× 1.49 NA oil immersion TIRF objective (Nikon), three 20-mW diode lasers (488 nm, 561 nm, and 640 nm), and an electron-multiplying charge-coupled device detector (iXon X3DU897; Andor Technology, Belfast, UK). Image acquisition was controlled using Nikon Elements software (version 5.02) and all assays performed for RT Images were acquired at 100 ms per frame for 200 frames. Maximum-intensity projections were generated, and kymographs were produced by drawing along tracks of motors (width = 3 pixels) using Fiji/ImageJ.

### 4.6. Golgi Dispersion Assays

The Golgi dispersion assay was adapted from a previously described protocol [33]. Briefly, U2OS cells were seeded onto glass coverslips and co-transfected with plasmids expressing a constitutively active, fluorescently tagged kinesin fused with a FKBP-rapamycin-binding (FRB) domain (e.g., KIF5C-1-560-mTagBFP-FRB) and a Golgi-targeting sequence (GMAP210) fused to mRFP and FKBP domains (GMAP210-mRFP-FKBP). Approximately 36 h after transfection, 44 nM of rapamycin was added to the media to induce heterodimerization between FRB and FKBP domain-containing proteins, thereby promoting the recruitment of FRB-tagged motors to Golgi-derived vesicles. After incubation for 30 min, the cells were washed with PBS and then fixed with 4% PFA in PBS. In some cases, pCDNA3 constructs encoding Flag-A51R or mutants thereof were also co-transfected. Coverslips were stained for Flag-tagged proteins via immunofluorescence as described [24]. Cells not treated with rapamycin were used as a control.

An Olympus Fv10i-LIV confocal microscope equipped with cellSens imaging software (version 1.18), a 60× water immersion objective, and 405, 473, 559, and 635 nm lasers were used in order to capture max-projection images of fixed cells to determine the ratio of mRFP signal in perinuclear areas to that in peripheral regions. Only cells with nuclei predominantly on one side of the cell were analyzed so that the major cytoplasmic space between the nuclear membrane and the plasma membrane could be equally divided into “perinuclear” and “peripheral” regions, and mRFP signals were measured using ImageJ.

### 4.7. Organelle Distribution Assay, B5R Staining, and StableMARK Imaging

For organelle distribution assays, HeLa cells were seeded at a density of 30,000 cells per coverslip, cultured overnight, and then infected with indicated VV strains. For lysosome staining, cells were treated with 200 nM of Lysotracker in media for 1 h before fixation with 4% paraformaldehyde (PFA). For mitochondria staining, cells were treated with 200 nM of Lysotracker in media for 30 min before fixation with PFA. After fixation, cells were incubated at RT with blocking buffer (PBS with 1% BSA and 0.1% Triton X-100) for 1 h, stained with rabbit anti-Flag primary antibody for 2 h, extensively washed with blocking buffer, incubated with Alexa Fluor-conjugated secondary antibody for 1 h, and then extensively washed with blocking buffer prior to mounting. Coverslips were mounted onto glass slides using ProLong^TM^ Diamond anti-fade with DAPI and imaged on an Olympus Fv10i-LIV confocal microscope equipped with cellSens imaging software (version 1.18). Similar imaging procedures were used for B5R staining experiments, except these were conducted in U2OS cells and used mouse anti-B5R antibody staining in conjunction with rabbit anti-Flag antibody staining.

For StableMARK imaging experiments, U2OS cells were seeded at a density of 30,000 cells per coverslip, cultured overnight, and then infected with indicated VV strains. For StableMARK transfections, U2OS were transfected with 2 μg of plasmid DNA using Lipofectamine 2000 (Invitrogen, Waltham, MA, USA) according to the manufacturer’s protocols in OptiMEM for 5–6 h. For cells that were both transfected and infected, cells were infected for 2 h and washed once with OptiMEM before performing the transfection. Cells were incubated for an additional 18 h after the media was replaced with complete culture media. Cells were then fixed with either methanol or 4% PFA, washed with PBS extensively, incubated at RT with blocking buffer for 1 h, stained with primary antibodies for 2 h, extensively washed with blocking buffer, incubated with Alexa Fluor-conjugated secondary antibodies for 1 h, and then extensively washed with blocking buffer prior to mounting. Coverslips were mounted onto glass slides using ProLong^TM^ Diamond anti-fade with DAPI and imaged on a Zeiss LSM980 Airyscan2 super-resolution microscope using a 40× oil objective with variable optical zoom and matched pixel size to achieve AiryScan2 super-resolution. The scope was equipped with monochromatic lasers (405/488/561 and 640) and Zeiss Zen blue imaging software (version 3.3); see figure legends for MOIs and time points used for imaging infected cells. References to “super-resolution” images in the manuscript indicate images taken with the Airyscan2 microscope to differentiate them from images captured with FV10i-LIV (confocal) or Abberior STED microscope (see below).

### 4.8. STED Microscopy

Samples imaged using STED microscopy (Figure S5B,D) were also prepared as described in Section 4.7 except the cells were seeded on 1.5 mm-thick coverslips and STAR RED- and STAR ORANGE-conjugated secondary antibodies were used for immunofluorescence staining. Coverslips were mounted onto glass slides using ProLong Diamond anti-fade (without DAPI). Single z plain 2D STED imaging was performed with the Facility Line of Abberior Instrument with Olympus 60× oil objective. Line mode was used to take dual-color STED images. STAR RED was imaged with a 640 nm excitation laser and a 775 nm depletion laser. STAR ORANGE was imaged with a 561 nm excitation laser and a 775 nm depletion laser. For Figure S5B, line scan mode was used to image STAR RED and STAR ORANGE with the following parameters: excitation of 640 nm 4% with 20% STED, 775 nm 40% with 15% STED. For Figure S5D, line scan mode was used to image STAR RED and STAR ORANGE with the following parameters: excitation of 640 nm 15% with 20% STED, 775 nm 25% with 15% STED. For all images, a dwell time of 5 μs and a pixel size of 10 nm were used. All images were taken with a 1.0 AU pinhole based on 640 nm.

### 4.9. Statistical Analyses

All statistical analyses were conducted using GraphPad Prism (v.8.0) software, and *p* values <0.05 were considered statistically significant. Sample sizes, statistical tests used, and relative *p* values are indicated in the respective figure or figure legend for quantitative experiments.

### 4.10. Key Reagent and Resources Sources

See Table S1 for source information regarding key reagents and resources used in this study.

## Data Availability

The original contributions presented in the study are included in the article/Supplementary Materials, and further inquiries can be directed to the corresponding author.

## Supplementary Materials

The following supporting information can be downloaded at: https://www.mdpi.com/article/10.3390/ijms25147825/s1. Reference [52] is cited in the Supplementary Materials.
