# Supplementary material for: Poxvirus A51R Proteins Negatively Regulate Microtubule-Dependent Transport by Kinesin-1
Source: Int J Mol Sci. 2024 Jul 17;25(14):7825. doi: 10.3390/ijms25147825 (PMC11277487; doi:10.3390/ijms25147825)
Supplement: Supplementary file 1 [file ijms-25-07825-s001.zip › ijms-3069934-supplementary.pdf]

Supplementary Information

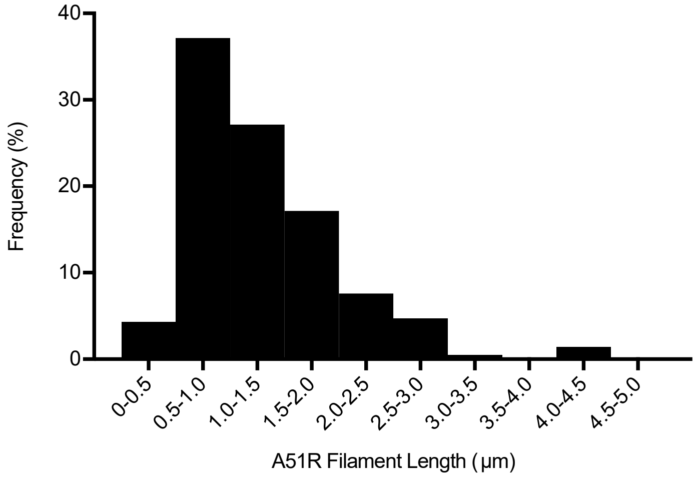

Figure S1. Length distribution of FA51R filaments on MTs from super-resolution imaging. Histogram illustrating frequency of MT-bound A51R filament lengths in U2OS cells infected with ΔA51R^{FA51R} for 18 h. A total of 428 A51R filaments were measured using CellSens software (Olympus).

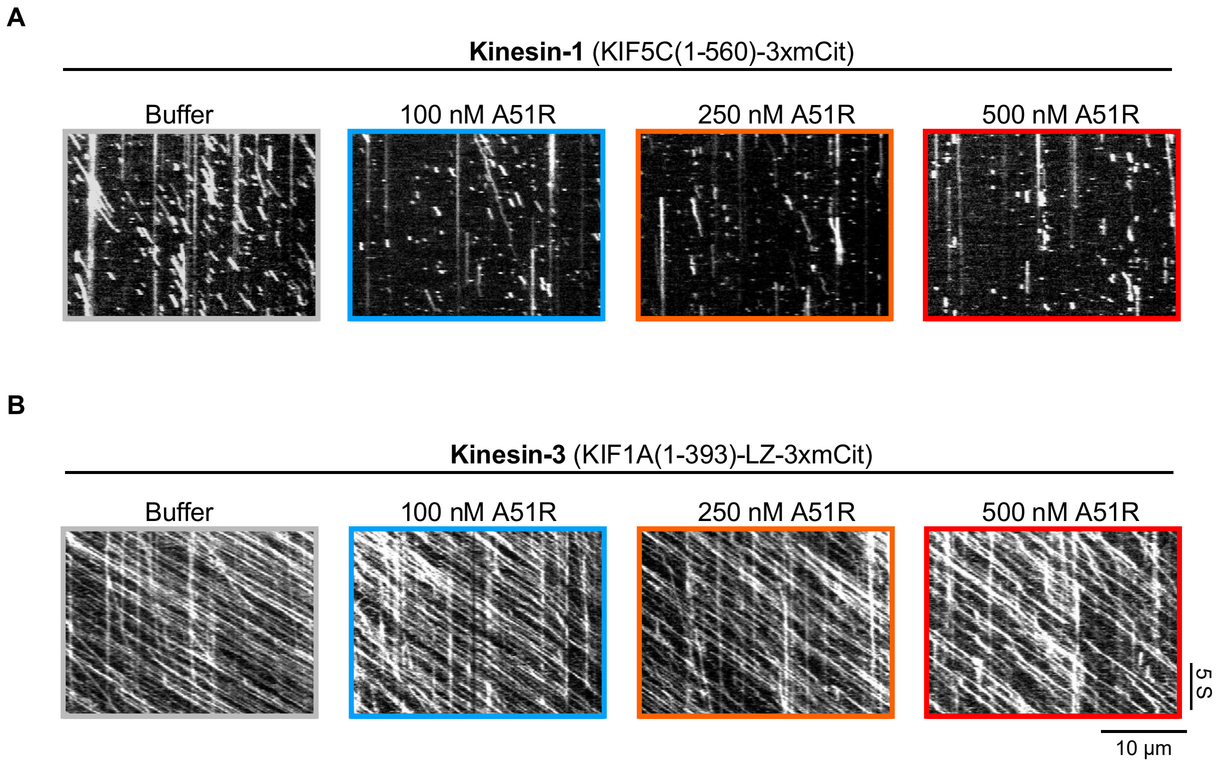

Figure S2. Representative kymographs used for quantitative analysis in single-molecule motility assays data in Figure 1 with indicated kinesin constructs.

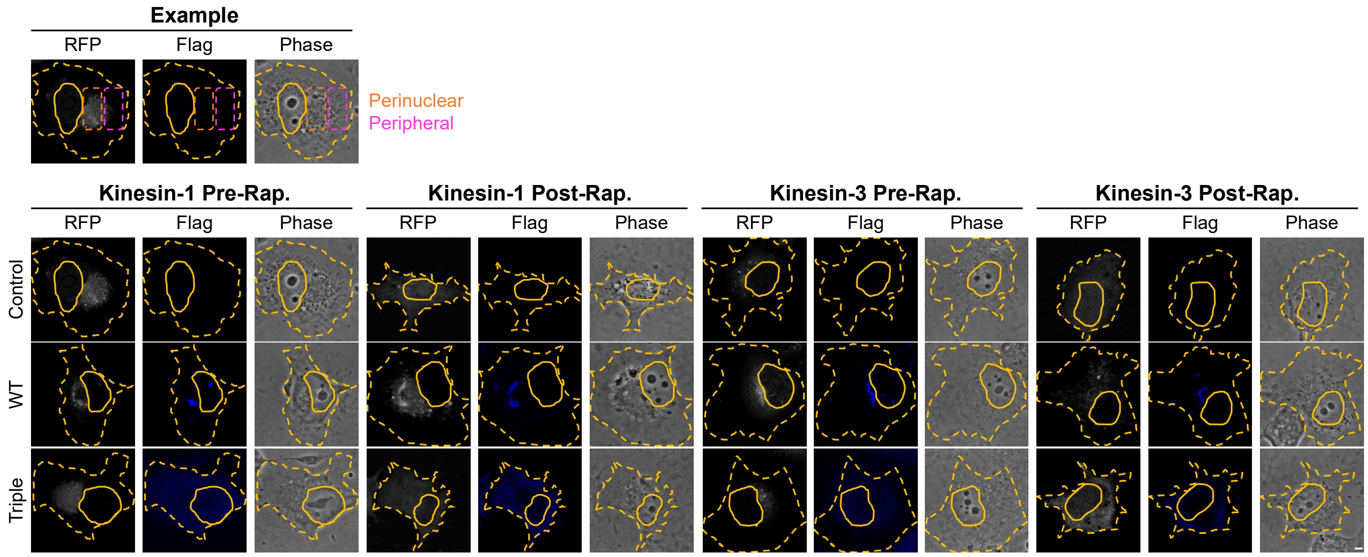

Figure S3. Representative confocal microscopy images from Golgi dispersions assays in Figure 2BC. Top panel: an example of how cell cytoplasm was equally divided into perinuclear and peripheral regions for RFP signal quantification. Dashed lines indicate cell edges. Solid lines indicate nucleus edges. Bottom Panel: Example images of cells used in quantification in Figure 2BC. Cells were co-transfected with indicated kinesin constructs along with Golgi-targeted RFP constructs empty vector (EV) or FA51R (WT) or FA51R^{Triple} (Triple) constructs. Scale bar = 5 μm. See Figure 2A legend for additional details.

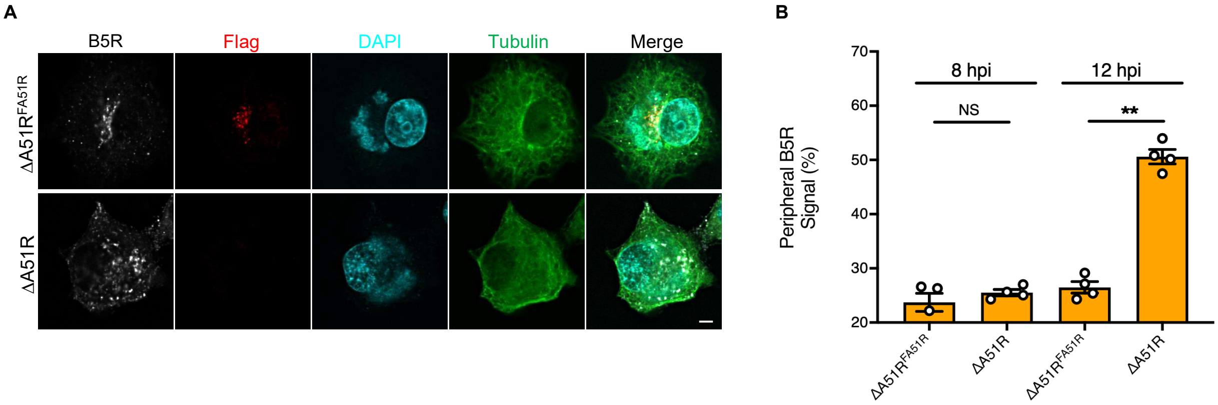

Figure S4. Differential IEV localization during $\Delta A51R^{FA51R}$ and ΔA51R infection. (**A**) Representative confocal microscopy images of $\Delta A51R^{FA51R}$- and ΔA51R-infected U2OS cells at 12 hpi (MOI = 3). B5R staining was used to mark IEV particles. Scale bar = 5 μm. (**B**) Distribution of B5R signal in $\Delta A51R^{FA51R}$- and ΔA51R-infected U2OS cells infected as in **A** for 8 or 12 hpi. Data are means (± SE). Unpaired two-tailed Student's t-test. ** = P<0.01, NS = not significant.

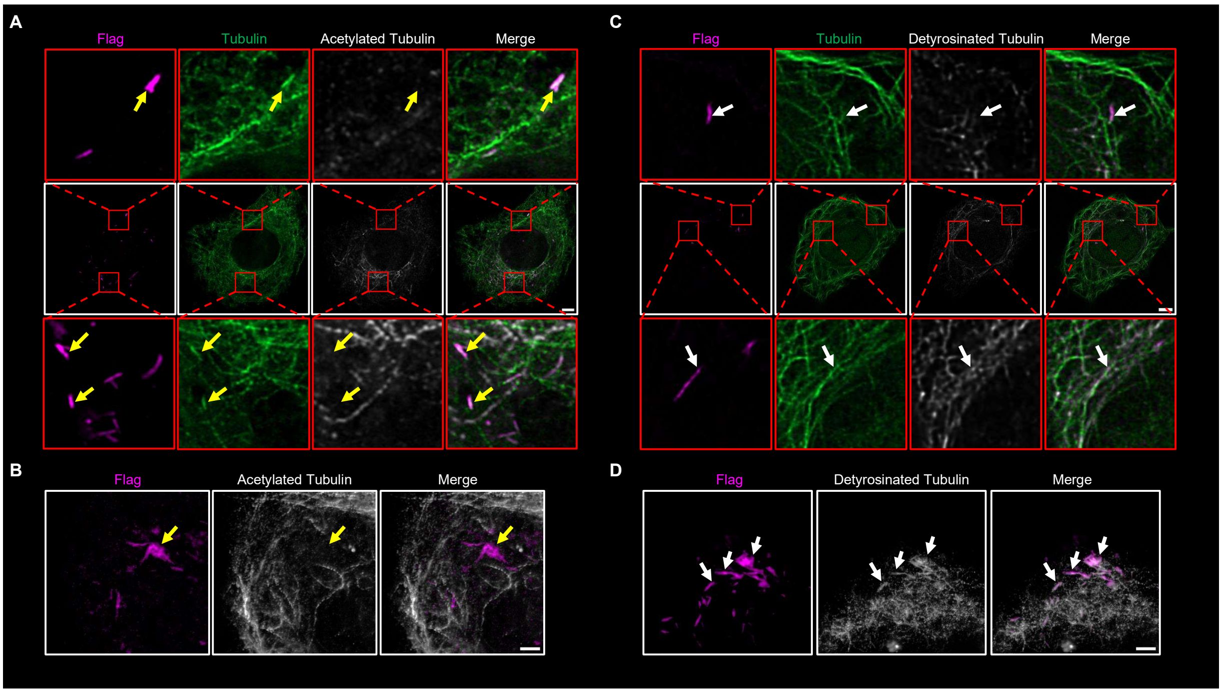

Figure S5. Analysis of A51R association with acetylated or detyrosinated MTs. (**A**) Representative super-resolution microscopy image of U2OS cell infected with ΔA51R^{FA51R} (MOI = 3) for 12 h and showing FA51R (Flag), total tubulin, and acetylated tubulin staining. Zoomed-in images of the areas indicated are shown with red boxes. Yellow arrows indicate A51R-bound MTs that are not visibly acetylated. Scale bar = 5 µm. (**B**) Representative STED microscopy image of U2OS cell infected with ΔA51R^{FA51R} (MOI = 3) for 12 h and showing FA51R (Flag) and acetylated tubulin staining. Scale bar = 2 µm. Yellow arrows indicate A51R-bound MTs that are not visibly acetylated. (**C**) Representative super-resolution microscopy image of U2OS cell infected with ΔA51R^{FA51R} (MOI = 3) for 12 h and showing FA51R (Flag), total tubulin, and detyrosinated tubulin staining. Zoomed-in images of the areas indicated are shown with red boxes. White arrows indicate A51R-bound MTs that are detyrosinated. Scale bar = 5 µm. (**D**) Representative STED microscopy image of U2OS cell infected with ΔA51R^{FA51R} (MOI = 3) for 12 h and showing FA51R (Flag) and detyrosinated tubulin staining. White arrows indicate A51R-bound MTs that are detyrosinated. Scale bar = 2 µm.

Table S1. Reagents and Resources.

REAGENT or RESOURCE	SOURCE	IDENTIFIER
Antibodies		
Rabbit polyclonal anti-Flag	Sigma-Aldrich	Cat# F7425, RRID:AB_439687
Mouse anti-α-Tubulin–FITC	Sigma-Aldrich	Cat# F2168, RRID:AB_476967
Mouse anti-α-Tubulin	Sigma-Aldrich	Cat# T6199, RRID:AB_477583
Rabbit polyclonal anti-Actin	Abcam	Cat# ab1801, RRID:AB_302617
Mouse anti-Vaccinia Virus I3L	Gammon et al. [52]	N/A
Mouse anti-L1R	BEI	NR-4511
Mouse anti-Vaccinia Virus B5R	BEI	NR-431
Mouse anti-acetyl-alpha tubulin, Clone 6-11B-1)	EMD millipore	MABT868
Rabbit monoclonal anti-detyrosinated alpha-tubulin, Clone RM444	RevMAb Biosciences	Cat# 31-1335-00
Donkey anti-Rabbit Secondary, Alexa Fluor 647	Invitrogen	Cat# A-31573
Donkey anti-Rabbit Secondary, Alexa Fluor 568	Invitrogen	Cat# A-10042
Goat anti-Mouse Secondary, STAR ORANGE	Abberior	STORANGE-1001
Goat anti-Rabbit Secondary, STAR RED	Abberior	STRED-1002
Virus Strains (alternative names in parentheses)		
$\Delta A51R^{FA51R}$	Gammon et al. [25]	N/A
$\Delta A51R^{FA51RTriple}$	Seo et al. [24]	N/A
E. cloni® Chemically Competent Cells	Biosearch Technologies	60108
BL21(DE3) cells	Agilent	200131
Chemicals, Peptides, and Recombinant Proteins		
Reporter Lysis 5X Buffer	Promega	E3971
HisTrap HP 1 ml column	GE Healthcare	17524701
Superdex 200 Increase 10/300 GL column	GE Healthcare	28990944
HIS-Select® Nickel Magnetic Agarose Beads	Sigma-Aldrich	H9914
Tubulin protein (> 99% pure) porcine brain	Cytoskeleton Inc.	T240
Tubulin protein (fluorescent HiLyte 647): porcine brain	Cytoskeleton Inc.	TL670M
GTP	Cytoskeleton Inc.	BST06
Paclitaxel (Taxol)	Sigma-Aldrich	T7402
Nocodazole	Sigma-Aldrich	M1404

Isopropylthio-β-galactoside (IPTG)	RPI	I560000
Imidazole	Sigma	I2399
cOmplete, EDTA-free Protease Inhibitor Cocktail	Sigma-Aldrich	11873580001
Phenylmethylsulfonyl Fluoride (PMSF)	Tocris Bioscience	44-865-0
ProLong Diamond Antifade Mountant with DAPI	Invitrogen	P36962
ProLong Diamond Antifade Mountant	Invitrogen	P36961
Dithiothreitol (DTT)	Fisher	BP172
Ethylenediaminetetraacetic acid (EDTA)	Fisher	BP2482
Dulbecco's Modified Eagle's Medium - high glucose	Sigma-Aldrich	D6429
Minimum Essential Medium Eagle	Sigma-Aldrich	M4655
Dulbecco's Phosphate Buffered Saline	Sigma-Aldrich	D8662
Antibiotic Antimycotic Solution (100x)	Sigma-Aldrich	A5955
MEM Nonessential Amino Acid Solution	Corning	25025CI
L-glutamine Solution	Corning	25005CI
FB Essence	Avantor Seradigm	3100
FBS	Atlanta Biologicals	S12450
Fetal Clone III	HyClone	SH30109
Glutamax	Gibco	35050061
Opti-MEM Reduced Serum Medium	Gibco	31985070
Lipofectamine 2000 Transfection Reagent	Invitrogen	11668019
Trans-IT LT1	Mirus	MIR 2304
LI-COR Odyssey Blocking Buffer	LI-COR	92740010
Amicon Ultra (centrifugal filters) 10 kD	Millipore	UFC901008
Amicon Ultra (centrifugal filters) 50 kD	Millipore	UFC905008
Rapamycin	Sigma-Aldrich	R8781
LysoTracker Deep Red	Invitrogen	L12492
MitoTracker Deep Red	Invitrogen	M22426
Experimental Models: Primary Cells and Cell Lines		
Human: Bone osteosarcoma epithelial cells (U2OS)	ATCC	HTB-96
Human: Cervical carcinoma cells (HeLa)	ATCC	CCL-2
Cercopithecus aethiops: Kidney fibroblast (COS-7)	ATCC	CRL-1651
Cercopithecus aethiops: Kidney epithelial cells (BSC40)	ATCC	CRL-2761
Recombinant DNA		
Plasmid: Flag-A51R pcDNA3	Gammon et al.[25]	N/A
Plasmid: Flag-A51R R275A/K295A/K302A pcDNA3	Seo et al. [24]	N/A
Plasmid: pET22b	Sigma	69744
Plasmid: His-A51R pET22b	Seo et al. [24]	N/A
Plasmid: hKif5B(1-560)rigor-2xmNeongreen pB80 (StableMARK)	Addgene	174649

Software and Algorithms		
ImageJ v1.51n	NIH	https://imagej.nih.gov/ij/
Image Studio v5.2	LI-COR Biosciences	https://www.licor.com/bio/image-studio-lite/
CellSens Imaging Software v1.18	Olympus	N/A
Bio-Rad Image Lab Software, v5.2.1	Bio-Rad	N/A
